# Effectiveness of Provider Education Followed by Computerized Provider Order Entry Alerts in Reducing Inappropriate Red Blood Cell Transfusion

**DOI:** 10.1155/2016/2859720

**Published:** 2016-12-05

**Authors:** Vijay M. Patel, Anna W. Rains, Christopher T. Clark

**Affiliations:** ^1^Department of Pathology, University of Tennessee Graduate School of Medicine, 1924 Alcoa Highway, Knoxville, TN 37920, USA; ^2^Department of Quality and Patient Safety, University of Tennessee Medical Center, 1924 Alcoa Highway, Knoxville, TN 37920, USA

## Abstract

To reduce the rate of inappropriate red blood cell transfusion, a provider education program, followed by alerts in the computerized provider order entry system (CPOE), was established to encourage AABB transfusion guidelines. Metrics were established for nonemergent inpatient transfusions. Service lines with high order volume were targeted with formal education regarding AABB 2012 transfusion guidelines. Transfusion orders were reviewed in real time with email communications sent to ordering providers falling outside of AABB recommendations. After 12 months of provider education, alerts were activated in CPOE. With provider education alone, the incidence of pretransfusion hemoglobin levels greater than 8 g/dL decreased from 16.64% to 6.36%, posttransfusion hemoglobin levels greater than 10 g/dL from 14.03% to 3.78%, and number of nonemergent two-unit red blood cell orders from 45.26% to 22.66%. Red blood cell utilization decreased by 13%. No additional significant reduction in nonemergent two-unit orders was observed with CPOE alerts. Provider education, an effective and low-cost method, should be considered as a first-line method for reducing inappropriate red blood cell transfusion rates in stable adult inpatients. Alerts in the computerized order entry system did not significantly lower the percentage of two-unit red blood cells orders but may help to maintain educational efforts.

## 1. Introduction

Red cell transfusion (RBC) has been shown to be associated with increased patient morbidity and mortality. RBC transfusion has been linked with increased duration of hospital stay, length of stay in the intensive care unit, duration on mechanical ventilation, risk of bacterial postoperative infection, and risk of multiple organ failure. Observational studies have shown this association to be dose-dependent [1–4]. In 2012, The Joint Commission and the American Medical Association-Convened Physician Consortium for Performance Improvement determined RBC transfusion to be one of the top five overuses in US medicine. It is important to note that the US transfuses more blood than other countries for comparable procedures without improved outcomes [5]. In 2012, AABB (formerly American Association of Blood Banks) published clinical guidelines for RBC transfusion based upon results and recommendations from 31 randomized clinical trials involving over 12 500 patients, comparing restrictive transfusion thresholds (7-8 g/dL) with liberal thresholds (9-10 g/dL). Although AABB could make no specific recommendation regarding liberal or restrictive transfusion strategy for patients with acute coronary syndrome, AABB recommends restrictive transfusion strategy in other stable, hospitalized patients. Transfusion is likely to be indicated in a patient with hemoglobin (Hgb) level less than 7 g/dL. AABB recommends a transfusion trigger where Hgb is less than 8 g/dL in patients with symptomatic preexisting cardiovascular disease. Transfusion is not likely to be indicated when Hgb level is greater than 10 g/dL [6]. The concept of liberal and restrictive transfusion strategy has been in the literature for some time. The Transfusion Requirements in Critical Care (TRICC) Trial in 1999 randomized 838 critical care patients into liberal (transfusion trigger of Hgb less than 10 g/dL with posttransfusion target Hgb of 10–12 g/dL) or restrictive (transfusion trigger of Hgb less than 7 g/dL with posttransfusion target Hgb of 7 to 9 g/dL) strategy. This trial reported that restrictive transfusion strategy is at least equivalent to liberal transfusion strategy in all groups, except for in patients with severe ischemic heart disease. The trial also reported that a restrictive transfusion strategy is potentially better in “less ill” (APACHE score less than 20) and younger (less than 55 years old) patients [7]. This has been supported in other reports, including a systematic review with meta-analyses and trial sequential analysis of 31 trails totaling 9813 randomized patients concluding restrictive transfusion strategies to be safe in most clinical situations. The authors also concluded that liberal transfusion strategies have not been shown to convey any benefit to patients [8]. Restrictive transfusion strategy is consistent with current evidence-based medicine. Education of ordering providers and adherence to restrictive strategy could reduce patient exposure to blood products and reduce potential risks associated with transfusion.

## 2. Materials and Methods

Our hospital system is a university-based teaching hospital with a Level I trauma center and neonatal intensive care unit. From 2014 through mid-2016, the computerized provider order entry (CPOE) system at our institution did not include orders related to massive transfusion protocol, intraoperative transfusion, outpatient transfusion, or orders from the neonatal intensive care unit. Since the AABB 2012 guidelines for RBC transfusion are for stable inpatients, we selected CPOE generated RBC transfusion orders as our mechanism of capturing our target patient population. Three main metrics were developed to align with AABB RBC transfusion guidelines and a restrictive transfusion strategy, including orders with a pretransfusion Hgb level greater than 8 g/dL, a posttransfusion Hgb level of greater than 10 g/dL, and nonemergent two-unit RBC orders. Nonemergent orders were defined as orders entered into CPOE, but orders that selected active bleeding or acute cardiovascular (CV) insufficiency as the transfusion indication were excluded. In April 2015, an official patient blood management program was established at our institution and a patient blood management safety coordinator was hired. An electronic database was established to obtain RBC orders entered into CPOE electronically on a daily basis and were reviewed in real time. CPOE orders were stratified by ordering provider and also by department. The hospitalist group, internal medicine resident staff, and family medicine resident staff were determined to have the highest order frequencies at our institution, respectively. Individual data for each of these departments and individual provider ordering information were obtained, graphed, and presented to each department. Each individual provider was given their personal transfusion practice relative to the established metrics, as well as the data for their own practice group. In addition, information was provided containing AABB RBC transfusion guidelines including verbal presentation and laminated pocket cards. Following this, the RBC transfusion orders were reviewed and email communications sent to the ordering provider when outside of metric indicator. If the provider was a resident physician, the email was also sent to the patient's attending physician of record. Copy of the AABB guidelines and review article regarding restrictive transfusion strategy was attached to all email communications. Additional literature was attached if specific information was available regarding a particular clinical situation. After six months, the data comparison before and after program metrics was represented to each individual group.

In April 2016, CPOE RBC transfusion alerts were activated. The alert is actionable, thereby allowing the ordering provider to continue the original order, cancel the order, or change the number of RBC units ordered for transfusion. The alert notifies the ordering provider that the order would likely be outside of AABB RBC transfusion guidelines and a restrictive transfusion strategy. Alert firing is based upon the order indication and pretransfusion Hgb, defined as most recent Hgb level within 24 hours of RBC order. Possible choices for RBC indication include active bleeding with hemodynamic instability, acute CV insufficiency, an Hgb level less than 7 g/dL in stable patients, an Hgb level less than 7.5 g/dL in stable ICU patients, an Hgb level less than 8 g/dL in non-ICU patients with symptomatic anemia, an Hgb level less than 8 g/dL in oncology patients with symptomatic anemia, and renal failure with symptomatic anemia. Alerts were developed in an attempt to target transfusion scenarios mirroring AABB 2012 guidelines for stable inpatients and limit provider alert fatigue. No alert will fire if the pretransfusion Hgb level is less than 6 g/dL. Since chronic renal failure patients and those undergoing chemotherapy are often transfused based upon clinical findings, no alert will fire if Hgb level was less than 8 g/dL with oncology or renal failure diagnosis. If the provider selects “active bleeding” or “acute CV insufficiency,” then no alerts will fire. When patients have active bleeding, the pretransfusion hemoglobin available in CPOE may not be reflective of the current clinical state or hemodynamic status. Patients with active bleeding are less likely to be stable inpatients. Available patient electronic medical records of orders that list “active bleeding” as the indication are reviewed retrospectively and email communications are sent to ordering providers in which the indication does not seem to fit the available information. In addition, AABB could make no transfusion recommendations for patients with acute coronary syndrome [6]. Otherwise, the alert will fire under the following conditions:

- One or more RBC units ordered for transfusion and an Hgb level greater than 7 g/dL without selected indication of active bleeding, acute CV insufficiency, oncology diagnosis, or renal failure
- Two or more RBC units ordered for transfusion and an Hgb level between 6 and 7 g/dL without selected indication of active bleeding, acute CV insufficiency, oncology diagnosis, or renal failure
- One or more units ordered and a pretransfusion Hgb level greater than 10 g/dL with selected indication of oncology diagnosis or renal failure
- Two or more units ordered and a pretransfusion Hgb level between 8 and 10 g/dL with selected indication of oncology diagnosis or renal failure

## 3. Results

Prior to the provider education program, the average rate in 2014 (12-month period) for RBC orders with a pretransfusion Hgb level greater than 8 g/dL was 16.64% and a posttransfusion Hgb level greater than 10 g/dL was 14.03% and for nonemergent two-unit RBC orders was 45.26%. From January through March of 2015, efforts were made to hire a patient blood management safety coordinator. The provider educational program officially began on April 1, 2015. After the initial 12 months of the program (April 2015 through March 2016), provider education resulted in the following metrics: the pretransfusion Hgb level greater than 8 g/dL was reduced to 6.36% (62% reduction, *p* value < 0.001), the posttransfusion Hgb level greater than 10 g/dL was reduced to 3.78% (73% reduction, *p* value < 0.001), and number of nonemergent two-unit red blood cell orders was reduced to 22.66% (50% reduction, *p* value < 0.001).

Compared to 2014, the total patient volume increased by 11% during the first 12-month period of the program (26430 annual adult inpatient admissions increased to 29404). Total percentage of inpatients transfused per 1000 patient days over the 12-month period decreased from 12.4% average in 2014 to 10.84% by March 2016 (13% reduction). The number of patients transfused per 1000 patient days decreased from 24.23% in 2014 to 21.26% (12% reduction). During this 12-month time period, the estimated RBC product acquisition cost savings are $130,000.

During the first 6 months of the program (April through September), there were 2925 RBC orders in CPOE, 784 RBC transfusion orders reviewed, and 117 email communications sent (15% of orders reviewed received email communications). During the second 6-month period (October through March), there were 2953 RBC orders in CPOE, 709 orders reviewed, and 81 e-mail communications sent (11% of orders reviewed). The percentage during months 10 through 12 (January through March), email communications were sent to 8% of orders reviewed.

**Figure 1:**
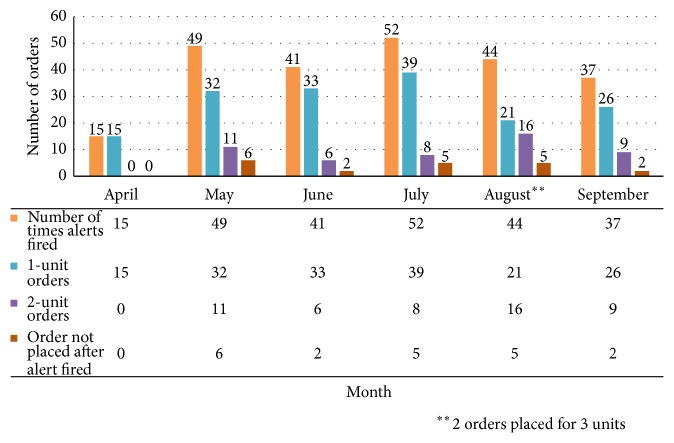
CPOE alert frequencies and actions.

**Figure 2:**
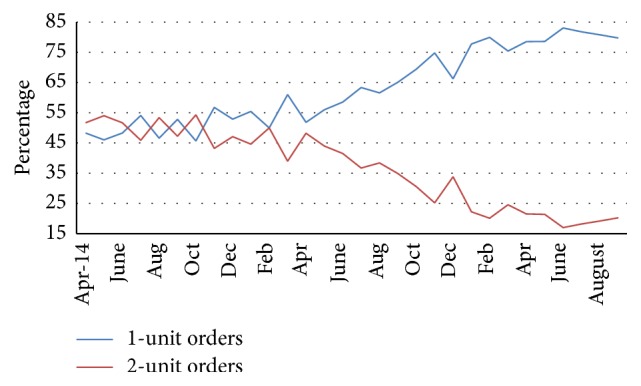
1-unit versus 2-unit RBC orders.

In April 2016, CPOE RBC alerts were activated. During the first 2-month period after implementation of CPOE RBC transfusion alerts, a flaw in the computer algorithm was in place and later corrected for June 2016. During the first 4-month period after corrected implementation of CPOE alerts (June through September 2016), the alerts fired 174 times (8% of total CPOE RBC orders). The order continued as a two-unit order 39 times (22%), the transfusion order was changed to one RBC unit 119 times (68%), and the order changed to no RBC units 14 times (8%) (Figure 1). Following implementation of corrected CPOE alerts for four months, rate for a pretransfusion Hgb level greater than 8 g/dL was 6.1%, a posttransfusion Hgb level greater than 10 g/dL was 4.47%, and nonemergent two-unit orders was 16.67%. There was no significant additional reduction in nonemergent two-unit RBC orders with implementation of CPOE alerts (Figure 2). However, of the 119 patients who had an order changed from two units to one unit, 84 patients (71%) did not receive any additional RBC units within 48 hours. The other 35 patients (29%), on average, received one additional RBC unit within 48 hours of the original order alert. The CPOE alerts prevented at least 98 RBC units (84 times two-unit order changed to one unit with no additional units ordered with 48 hours and 14 times when the RBC order was cancelled) from being transfused within a 4-month time period. This alone is an acquisition cost savings of $16,600. During this same time period, 490 RBC transfusion orders were reviewed with 36 email communications sent to ordering providers (7% of orders reviewed, 1.7% of total CPOE RBC orders).

**Figure 3:**
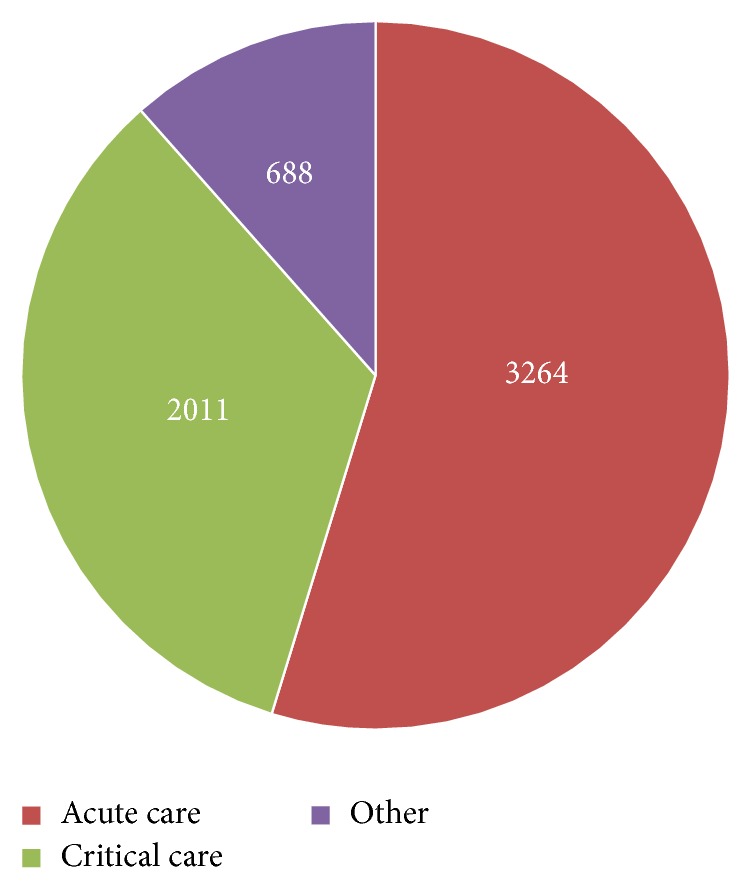
Patient location (Jan–Dec 2014), prestudy group.

**Figure 4:**
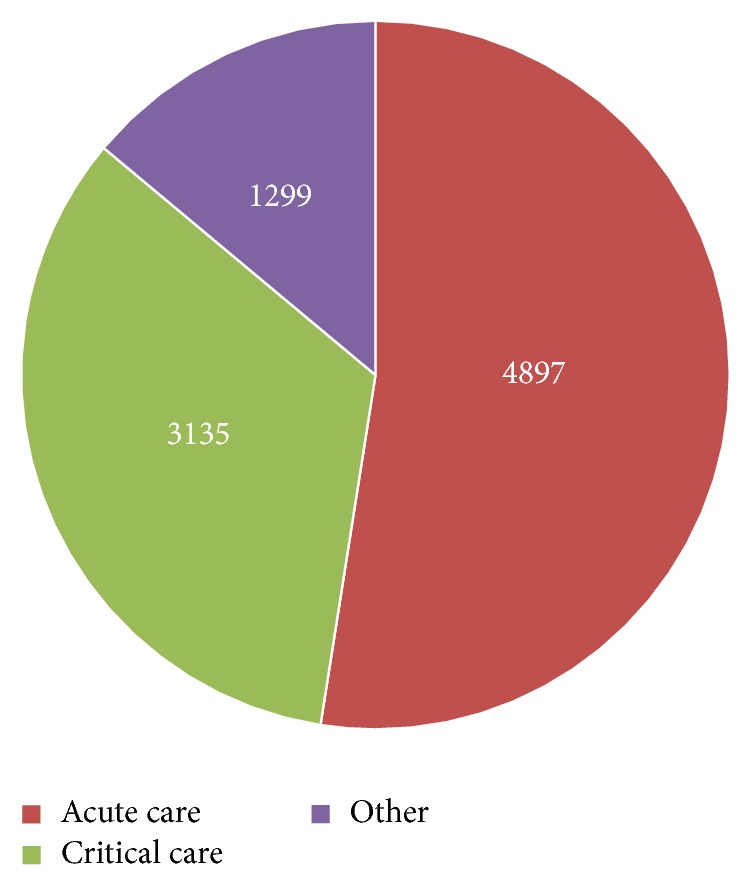
Patient location (April 2015–Sep 2016), study group.

The case mix by patient location of the prestudy group (Figure 3) and that of study group (Figure 4) patient populations are similar. The prestudy group included 5963 CPOE RBC transfusion orders: 3264 (54.7%) from acute care locations, 2011 (33.7%) from critical care locations, and 688 (11.5%) from other locations. The study group included 9331 CPOE RBC transfusion orders: 4897 (52.5%) from acute care locations, 3135 (33.6%) from critical care locations, and 1299 (13.9%) from other locations. In addition, the distribution of male and female patients among locations within the study groups is similar (Table 1).

## 4. Discussion

Previous studies have shown a reduction in inappropriate transfusion by various intervention techniques [9–14]. Prior to the AABB 2012 RBC transfusion guidelines, previous studies have been challenged with defining inappropriate transfusions. In addition, many studies do not report case mix of prestudy and poststudy patient groups. In 2002, Wilson et al. reviewed nine published articles reporting effectiveness of interventions to reduce inappropriate transfusion. While the definitions for inappropriate transfusion varied and the authors used a variety of intervention techniques, Wilson et al. concluded that there is adequate evidence to support behavioral interventions to decrease inappropriate transfusion practice among health care providers [12]. In 2005, Tinmouth et al. reported similar conclusions after reviewing 19 published studies on behavioral interventions and physicians' transfusion practices [14].

In our program, provider education utilizing AABB 2012 RBC transfusion guidelines was implemented as a component of the overall strategy to decrease inappropriate RBC transfusion. In order to accomplish this goal, a program coordinator was needed to help implement the process and for data retrieval and review. While hiring personnel during the current economic climate was challenging, preliminary data demonstrating the proposed metrics and current state in 2014 were presented, along with financial data, to hospital senior management. Only a 5% reduction in inpatient RBC utilization would be cost neutral in hiring the coordinator. During the first 12-month period of the program, a 13% reduction in the target population was achieved, far exceeding the cost of the program coordinator. The institution also greatly benefited from the additional activities of the coordinator who was also actively involved in patient safety, review of reported potential adverse transfusion reactions, review of transfusion policies/procedures, review of training modules for nursing staff, performing direct observations of blood administration, providing educational lectures/materials to ordering providers/nursing staff, assisting with implementation of clinical pathways involving transfusion, and serving as transfusion resource throughout the institution.

We implemented real-time provider communication and feedback via email, immediately following educational lectures. Handouts containing AABB 2012 RBC transfusion guidelines and literature regarding the restrictive RBC transfusion strategy were distributed throughout the hospital system. Following one year, we implemented CPOE alerts in attempt to further decrease inappropriate use and to help sustain the educational efforts. Tavares et al. reported that physician education alone is desirable but may not greatly decrease RBC usage. However, they did report that, with prospective physician notification for potential modification, they achieved an approximate 33% decrease in RBC usage from baseline over a 3-year period without evidence of change in mortality [15]. Some reports noted success with decreased RBC utilization with CPOE alerts alone. Yerrabothala et al. in 2014 reported that the use of prospective computerized order auditing (CPOE) alone significantly reduced number of RBC units transfused per 1000 patient-days (60.8 to 44.2, *p* < 0.0001). They also noted that the proportion of two-unit RBC orders decreased from 47% to 15% [16]. Zuckerberg et al. in 2015 reported a provider education program which was followed by CPOE algorithms (clinical decision support). They noted that, with education alone for all surgical services combined, RBC utilization decreased by 16.4%. However, with CPOE algorithms the overall decrease was 14.3%. They report that adding CPOE and clinical decision support did not further reduce RBC utilization and suggested education as an effective measure to reduce RBC utilization [17]. Our program only included adult inpatients. RBC transfusions that occurred during emergent transfusion, active bleeding, or during surgery were excluded, aligning with AABB 2012 RBC transfusion guidelines for stable adult inpatients.

We initially changed the CPOE order to default to one unit and required multiple additional steps to complete an RBC order for more than one unit. However, with this strategy, we saw no change in practice. The additional steps did not deter the ordering provider from successfully ordering inappropriately. The educational efforts showed the most dramatic reductions in inappropriate RBC transfusion; however, the CPOE alerts were implemented following one year of educational efforts. With the recent implementation of CPOE within our system and concerns of “alert fatigue,” the educational program was initiated prior to the activation of alerts. With this strategy, the alerts are likely now more meaningful to the ordering providers and more directed at providers resistant to change following the educational program. In addition, the education program and CPOE alerts decreased the number of orders triggering manual review for appropriateness. While the implementation of CPOE alerts did not show additional reduction in our nonemergent two-unit RBC orders, we feel they are important to help encourage and maintain a restrictive transfusion strategy following the education efforts. This is supported by the fact that over 70% of the patients who had an order changed from two units to one unit following a CPOE alert did not receive any additional RBC units within 48 hours of the order alert.

AABB published updated RBC transfusion guidelines in October 2016. In the update, AABB proposes an expanded two-tier recommendation for most inpatients, but states evidence supports transfusion decision to be within the clinical context and to also consider patient preferences and available alternatives to transfusion. The updated guidelines recommend restrictive transfusion strategy with transfusion not indicated until an Hgb level less than 7 g/dL in hospitalized adult patients who are hemodynamically stable, including those who are critically ill. AABB recommends a restrictive threshold of 8 g/dL for patients undergoing orthopedic surgery and cardiac surgery and those with preexisting CV disease. They do state that restrictive transfusion threshold is likely comparable between 7 and 8 g/dL. Again, AABB states that the recommendations do not apply to acute coronary syndrome patients, as well as to patients with severe thrombocytopenia and chronic transfusion-dependent anemia due to insufficient available evidence. Another addition to the update is inclusion of the age of product at transfusion. AABB states that RBC units can be selected at any point of their licensed dating period and does not recommend limiting selected patient populations to only fresh (less than 10 days) RBC units [18]. As with all areas of medicine, the field of transfusion is evolving. With the updated 2016 AABB RBC guidelines, the CPOE alerts can be further fine-tuned to incorporate the most recent evidence-based recommendations. While CPOE alerts were activated based upon 2012 AABB guidelines, the updated guidelines state that the clinical context is important in the decision to transfusion. Active review of the CPOE alert orders supports that the ordering provider's judgment within the clinical context still plays an important role in the care of patients. Many factors are involved in oxygenation, not just the Hgb level. Therefore, future studies and recommendations may include additional parameters to better access oxygenation and allow for more streamlined transfusion decision-support algorithms.

Our study has several strengths. We have a relatively large sample size of prestudy and study group RBC transfusion orders. Our overall case mix of acute care/critical care patients and male/female ratios are very similar in both groups. We were able to define a specific target group of stable adult inpatients which directly mirror the patient population of the 2012 AABB guidelines. Our CPOE alert definitions not only were developed consistently with 2012 AABB guidelines, but also demonstrate consideration of ordering provider fatigue that could potentially decrease the effectiveness of the alerts. We also developed a metric of a posttransfusion Hgb level greater than 10 g/dL in order to not only monitor the pretransfusion trigger but also support restrictive posttransfusion target strategy. However, we could not define the patient population case mix stratified by comorbidities which potentially could have an impact on transfusion practice. While AABB guidelines for platelet transfusion are available [19], our study only addresses RBC transfusion practice and our patient population only includes stable adult hospitalized patients.

## 5. Conclusions

Provider education is an effective and low-cost method and should be considered as a first-line method to reduce inappropriate red blood cell transfusion rate in hospitalized adult patients. Following an effective education program, alerts in CPOE may not further decrease inappropriate transfusion significantly but may help to encourage and maintain restrictive transfusion strategy following provider education.

## Figures and Tables

**Table 1:** Distribution of male and female patients by location.

Location/Gender	Pre-Study Group (Jan–Dec 2014)		Study Group (Apr 2015–Sep 2016)	
	Total	% of Total	Total	% of Total
Acute Care	**3264**	**54.7%**	**4897**	**52.5%**
Female	1861	57.0%	2788	56.9%
Male	1403	43.0%	2109	43.1%
Critical Care	**2011**	**33.7%**	**3135**	**33.6%**
Female	921	45.8%	1329	42.4%
Male	1090	54.2%	1806	57.6%
Other	**688**	**11.5%**	**1299**	**13.9%**
Female	358	52.0%	678	52.2%
Male	330	48.0%	621	47.8%
Grand Total	**5963**	**100.0%**	**9331**	**100.0%**
Female	3140	52.7%	4536	48.6%
Male	2823	47.3%	4795	51.4%
